# Proton Pump Inhibitors Reduce Pancreatic Adenocarcinoma Progression by Selectively Targeting H^+^, K^+^-ATPases in Pancreatic Cancer and Stellate Cells

**DOI:** 10.3390/cancers12030640

**Published:** 2020-03-10

**Authors:** Marco Tozzi, Christiane E. Sørensen, Lara Magni, Nynne M. Christensen, Rayhana Bouazzi, Caroline M. Buch, Matteo Stefanini, Claudia Duranti, Annarosa Arcangeli, Ivana Novak

**Affiliations:** 1 Section for Cell Biology and Physiology, Department of Biology, University of Copenhagen, 2100 Copenhagen, Denmark; marco.tozzi@sund.ku.dk (M.T.); ches@sund.ku.dk (C.E.S.); lara.magni@bio.ku.dk (L.M.); nmchristensen@bio.ku.dk (N.M.C.); rayhanabouazzi@hotmail.com (R.B.); carolinembuch@gmail.com (C.M.B.); 2 Section of Clinical Oral Microbiology, Department of Odontology, Faculty of Health and Medical Sciences, University of Copenhagen, 2200 Copenhagen, Denmark; 3 Department of Experimental and Clinical Medicine, Section of Internal Medicine, University of Florence, 50134 Florence, Italy; matteo.stefanini@unifi.it (M.S.); Claudia.duranti@unifi.it (C.D.); Annarosa.arcangeli@unifi.it (A.A.); 4 DI.V.A.L. Toscana SRL, 50019 Sesto Fiorentino, Florence, Italy

**Keywords:** pancreatic cancer, pH regulation, K^+^ channels, pantoprazole, pancreatic stellate cells, fibrosis, cyclin D1, STAT3, PDAC, P-CAB

## Abstract

Pancreatic duct cells are equipped with acid/base transporters important for exocrine secretion. Pancreatic ductal adenocarcinoma (PDAC) cells may utilize such transporters to acidify extracellular tumor microenvironment, creating a niche favoring cell proliferation, fibrosis and resistance to chemotherapy—all contributing to the notoriously bad prognosis of this disease. Here, we report that gastric and non-gastric H^+^, K^+^-ATPases (coded by *ATP4A* and *ATP12A*) are overexpressed in human and murine pancreatic cancer and that we can target them specifically with proton pump inhibitors (PPIs) and potassium-competitive acid blockers (P-CABs) in in vitro models of PDAC. Focusing on pantoprazole, we show that it significantly reduced human cancer cell proliferation by inhibiting cellular H^+^ extrusion, increasing K^+^ conductance and promoting cyclin D1-dependent cell cycle arrest and preventing STAT3 activation. Pantoprazole also decreased collagen secretion from pancreatic stellate cells. Importantly, in vivo studies show that pantoprazole treatment of tumor-bearing mice reduced tumor size, fibrosis and expression of angiogenic markers. This work provides the first evidence that H^+^, K^+^-ATPases contribute to PDAC progression and that these can be targeted by inhibitors of these pumps, thus proving a promising therapeutic strategy.

## 1. Introduction

Pancreatic ductal adenocarcinoma (PDAC), the predominant form of pancreatic cancer, is among the leading cancer-related death causes with a five-year survival rate of about 9% [1]. Despite knowledge about the epidemiology and genetics of the disease [2,3,4], our understanding of the cellular and molecular mechanisms that give rise to PDAC is inadequate and limits development of novel, urgently needed, therapeutic approaches.

PDAC is a solid tumor, rich in stromal cells, exhibiting marked fibrosis, stiffness and poor vascularization [2,5]. Solid tumors display hypoxia and altered pH regulation due to disruption of normal intra- to extracellular pH gradients, resulting in acidification of the extracellular tumor microenvironment [6]. Recently, the extracellular acidity and adverse conditions in the tumor microenvironment have become recognized as important contributors to malignancy, resistance to chemotherapy, invasiveness and protection of transformed cells from clearance by the immune system [7,8]. Cancer cells are highly metabolically active, and take up large amounts of glucose, which they largely convert to lactic acid, even in presence of oxygen and functional mitochondria, (as described by the Warburg effect) [7,9,10]. This leads to considerable cellular production of protons that are extruded from the cells and acidify the local extracellular environment [9]. The required net acid export is accomplished by acid/base transporting membrane proteins, whose expression can be altered in malignantly transformed cells. Among these are lactate-H^+^ cotransporters, Na^+^/H^+^-exchangers, bicarbonate transporters of the SLC26 or SLC4 families and proton pumps, such as the vacuolar H^+^-ATPase (V-ATPase) and the gastric and non-gastric H^+^,K^+^-ATPases [6]. Dysfunctional and dysregulated acid/base transport proteins may thus constitute novel potential therapeutic targets in cancer pathologies such as PDAC.

In health, pancreatic duct epithelial cells secrete highly alkaline, bicarbonate-rich fluid, which is accomplished via luminal bicarbonate transport, balanced by basolateral export of protons into the interstitial extracellular environment [11,12]. To achieve this function, while maintaining intracellular pH homeostasis, pancreatic duct cells are equipped with specific, tightly regulated, acid/base transporters [12]. This special feature leaves pancreatic duct cells well-adapted to survival in a milieu characterized by pH fluctuations, which, under pathological conditions such as cancer, also leaves them pre-adapted to the selection pressure present in the acidified extracellular environment of PDAC [13]. This may be a crucial factor that contributes to the aggressiveness, invasiveness and high chemotherapy resistance of PDAC.

Human and rodent pancreatic ductal epithelial cells express functional gastric and non-gastric H^+^, K^+^-ATPases, which contribute to secretin-stimulated secretion and intracellular pH regulation [11,14,15]. However, it is unknown whether these pumps play a role in pancreatic pathologies, particularly in PDAC, in which dysfunctional pH regulation may contribute to the development, progression and severity of the disease. Gastric and non-gastric H^+^,K^+^-ATPases can be specifically targeted by proton pump inhibitors (PPIs), such as omeprazole and pantoprazole, and by potassium-competitive acid blockers (P-CABs), such as SCH-28080 [16,17]. The effect of PPIs on tumor development has been investigated in several studies on different types of cancer models [18,19,20,21]. However, except for the study on gastric cancer cells [20], most reports conclude that the effects of PPIs were on the V-ATPase. 

Therefore, in the present study we addressed the hypothesis that the gastric and non-gastric H^+^, K^+^-ATPases of pancreatic ducts could be involved in PDAC development and that PPIs could affect tumor growth both in vitro and in vivo.

## 2. Results

### 2.1. H^+^,K^+^-ATPase Subunits are Expressed in PDAC Cells and Murine and Human Pancreatic Cancer

**Figure 1:**
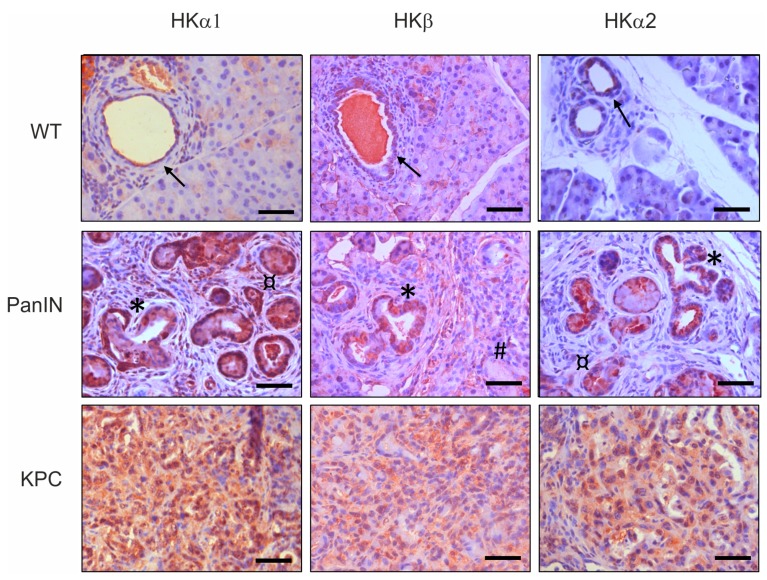
**Localization of H^+^, K^+^-ATPases subunits in mouse models of pancreatic cancer.** Samples prepared from paraffin embedded tissue from wild type mice (WT), Pdx-1-Cre, LSL-Kras^G12D/+^ double-transgenic mice (PanIN) and Pdx-1-Cre,LSL-Kras^G12D/+^,LSL-Trp53^R172H/+^ triple-transgenic mice (KPC). Immunohistochemistry for HKα1 stained with Calbiochem 119101 (1:200), HKα2 labeled with C384-M79 (1:200) and HKβ stained with Sigma A274 (1:200). Symbols indicate respectively: → pancreatic ducts, * PanIN structures, ¤ acino-ductal metaplasia (ADM), # mucous cells. Scale bars indicate 30 μm.

**Figure 2:**
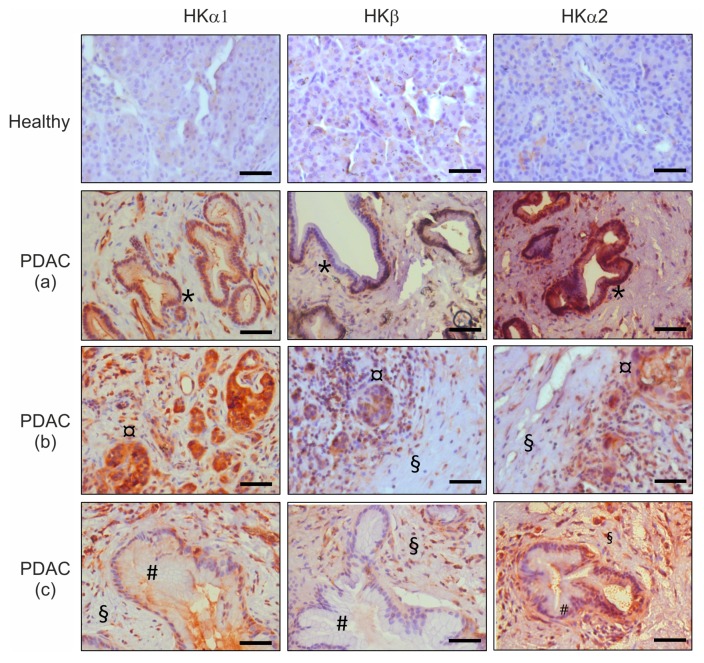
**Localization of H^+^, K^+^-ATPases subunits in human PDAC.** Samples prepared from human pancreatic tissues showing immunohistochemical reaction for HKα1 stained with Calbiochem 119101 (1:100), HKβ stained with Sigma A274 (1:200) and HKα2 labeled with C384-M79 (1:100). Top panel shows non-cancerous “healthy” tissue. PDAC (a,b,c) panels show different areas of the pancreas where the following can be distinguished: PanIN structures (*), acino-ductal metaplasia (ADM) (¤), mucous cells (#), pancreatic stellate cell (PaSC)-fibrosis (§). Scale bars indicate 30 μm.

We evaluated the expression pattern of H^+^, K^+^-ATPase subunits in murine and human pancreas samples (Figure 1 and Figure 2). Pdx-1-Cre, LSL-Kras^G12D/+^ double mutant mice show pre-cancerous pancreatic intraepithelial neoplasias (PanIN), and Pdx-1-Cre,LSL-Kras^G12D/+^, LSL-Trp53^R172H/+^ (KPC) triple mutant transgenic mice develop the invasive pancreatic ductal adenocarcinoma [22,23]. Pancreatic tissue from both the pre-cancerous PanIN lesions and KPC mice showed pronounced positive staining and high expression of all three H^+^,K^+^-ATPases subunits in ductal and in tumor cells (Figure 1). These are: the gastric α subunit (HKα1) and the β subunits (HKβ) of H^+^,K^+^-ATPases; and the non-gastric α subunit (HKα2) of H^+^,K^+^-ATPases (coded by *ATP4A*, *ATP4B* and *ATP12A*, respectively). The non-gastric HKα2 can borrow the gastric β subunit or one of the β subunits from the Na^+^,K^+^-ATPase for the pump assembly. Tissue sections with PanIN lesions display a large proportion of stroma, and neoplastic ducts exhibit morphology that differs from the normal pancreatic duct structure. Additionally, mucous structures and acino-ductal metaplasia (ADM) were detected. Staining for H^+^, K^+^-ATPase subunits was markedly increased and epithelial-like localization of, for example, HKα1 was no longer apparent. Notably, also stromal cells, most likely corresponding to pancreatic stellate cells (see below), were strongly stained. Stromal infiltration and lack of organotypic structure are further seen in the tissue sections with fully developed PDAC stage from KPC mice, in which the normal epithelial cells are replaced by neoplastic cells and extended areas of fibrosis and necrosis. 

Importantly, H^+^, K^+^-ATPase expression patterns in human pancreas (“healthy” tissue vs. cancer tissue taken from Whipple resection) (Figure 2) were similar to the ones in mice. Supplementary Figure S1 shows more clearly cellular localization of HK subunits in human ducts and PanIN lesions. Altogether, Figure 1 and Figure 2 show that all HK subunits were strongly expressed in the different stages of PDAC tissue in both mice and humans. 

**Figure 3:**
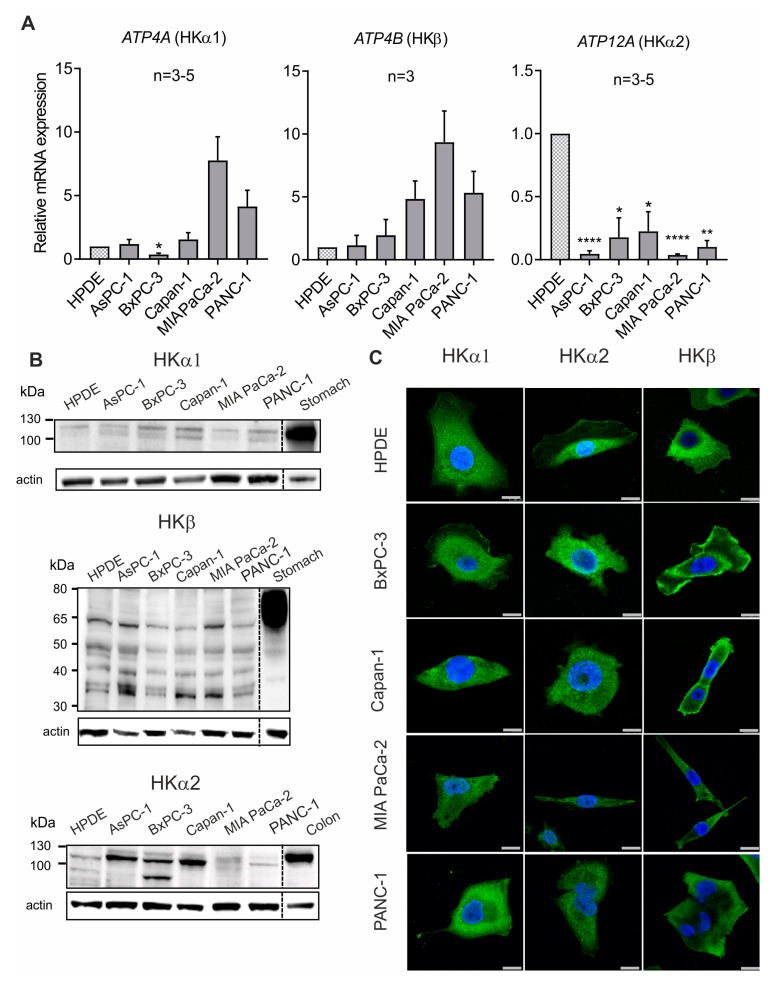
**Expression of H^+^, K^+^-ATPase subunits HKα1, HKα2 and HKβ in human pancreatic duct cell lines.** (**A**) Quantitative RT-PCR showing the mRNA expression of the three subunits relative to HPDE. Data are shown as means ± s.e.m. from indicated number of independent experiments. One-sample t-test with Holm-Bonferroni correction was used for statistic: *ATP4A*: * *p* = 0.0145, *ATP12A*: **** *p* < 0.0001; ** *p* = 0.0012 and * *p* = 0.0116 and 0.0151. Unadjusted *p* value for *ATP4B* in PANC-1 and MIA PaCa2 were 0.0473 and 0.0280. (**B**) Representative Western blot for HKα1 subunit (Abcam EPR12251), HKα2 subunit (Sigma, HPA039526) and HKβ subunit (Sigma A274) on the non-cancer cell line HPDE, and PDAC cell lines AsPC-1, BxPC-3, Capan-1, MIA PaCa-2 and PANC-1, as well as control tissues (mouse stomach and colon). Loading control was β-actin. (**C**) Immunolocalization of HKα1 (Calbiochem 119101, 1:100), HKα2 (C384-M79, 1:300) and HKβ (Sigma A274, 1:100) is reported in green. DAPI (blue) was used to stain the nuclei. Scale bars indicate 25 μm.

Next, we determined the expression of the H^+^,K^+^-ATPases subunits in five different human pancreatic ductal adenocarcinoma cell lines (AsPC-1, BxPC-3, Capan-1, MIA PaCa-2, PANC-1) (Figure 3A–C). The human “normal” pancreatic duct epithelial cell line HPDE was used as an example of non-cancer cell line. Similar to our earlier studies [14,15], we performed quantitative PCR on all cell lines used (Figure 3A). It appears that in the PDAC cells the gastric ATPase subunits (HKα1 and HKβ) expression seemed enhanced in some PDAC cell lines compared to HPDE cells, while the non-gastric ATPase α subunit was more prominent in HPDE cells. Expression was also confirmed on a protein level (Figure 3B). Protein extracts from mouse stomach and colon were used as positive controls for gastric and non-gastric H^+^, K^+^-ATPases, respectively. HKα1 and HKα2 are expressed as doublets around 110 kDa. Similar results have previously been shown in the rat pancreas, stomach, kidney and colon [14]. The gastric HKβ subunit is usually a highly glycosylated protein in the mouse stomach. In all cell lines, we detected HKβ bands of various sizes, including the core protein of 35 kDa, variably glycosylated forms and the full-glycosylated form around 60 kDa. Collectively, our expression data from the human PDAC and HPDE cells are in agreement with previously published observations of H^+^, K^+^-ATPases expression in human pancreatic ducts and two other PDAC cell lines [15]. We further assessed the cellular localization of the three different subunits by immunocytochemistry in HPDE and PDAC cells. HKα1-, HKα2- and HKβ-subunit fluorescence was detected on or close to the plasma membrane and in the intracellular compartment (Figure 3C). 

### 2.2. PPIs and P-CABs Inhibit PDAC Cell Proliferation

**Figure 4:**
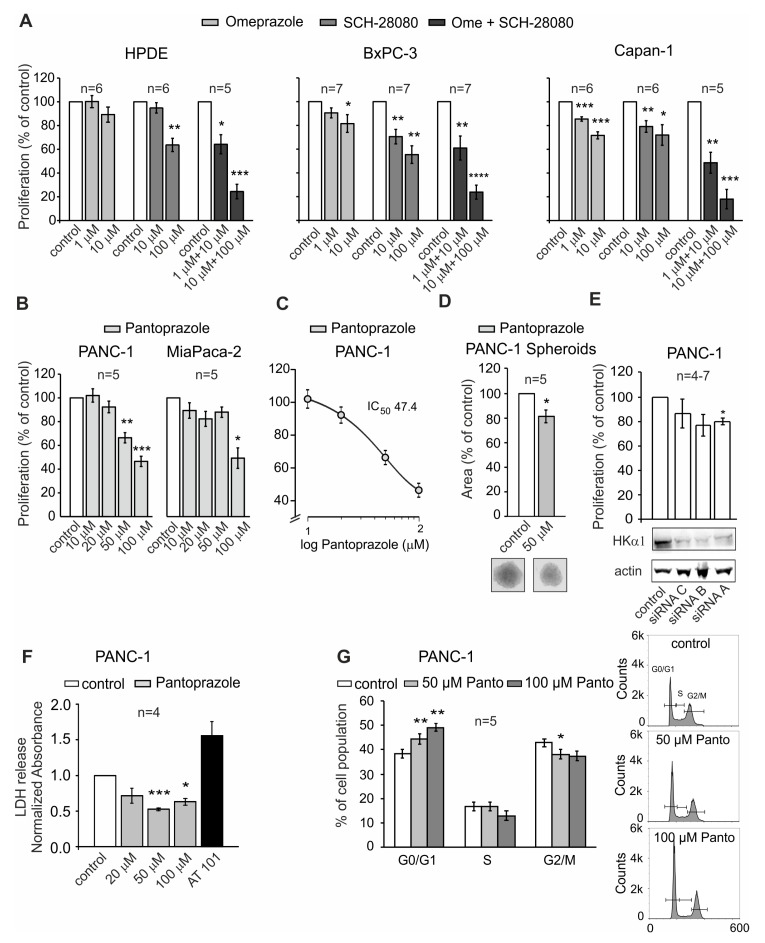
**Role of H^+^, K^+^-ATPase in PDAC cell proliferation, cell viability and cell cycle.** (**A**) Effect of PPIs and P-CAB on cell proliferation in HPDE (100 µM SCH-28080: ** *p* = 0.0013; 1 µM Ome+10 µM SCH-28080:* *p* = 0.0114; 10 µM Ome+100 µM SCH-28080: *** *p* = 0.0002), BxPC-3 (10 µM Ome: *P= 0.0485; 10 µM SCH-28080: ** *p* = 0.003; 100 µM SCH-28080: ** *p* = 0.001; 1 µM Ome+10 µM SCH-28080: ** *p* = 0.0084; 10 µM Ome+100 µM SCH-28080: **** *p* < 0.0001) and Capan-1 (1 µM Ome: *** *p* = 0.0006; 10 µM Ome: *** *p* = 0.0002; 10 µM SCH-28080: ** *p* = 0.0083; 100 µM SCH-28080: * *p* = 0.0233; 1 µM Ome+10 µM SCH-28080: ** *p* = 0.0042; 10 µM Ome+100 µM SCH-28080: *** *p* = 0.0006) cell lines; one-sample t-tests. Cells were incubated for 24 h with two different concentrations of omeprazole and SCH-28080, individually and together. (**B**) The effect of various concentrations of pantoprazole on proliferation of PANC-1 (50 µM:** *p* = 0.0042; 100 µM:*** *p* = 0.0008) and MIA PaCa-2 cells (100 µM: * *p* = 0.0168), one-sample t-tests and *p* values adjusted for multiple comparisons as more than two different conditions were tested against controls. (**C**) Dose-response curve for pantoprazole on PANC-1 cell proliferation. (**D**) Effect of pantoprazole treatment on PANC-1 spheroid sizes (* *p* = 0.0273 (one-sample t-test)). (**E**) Effect of three different siRNAs targeted to HKα1 on PANC-1 cell proliferation (* *p* = 0.012) with respective western blot showing the effectiveness of the siRNAs on HKα1 protein expression. (**F**) Effect of various concentrations of pantoprazole on LDH (lactate dehydrogenase) release in PANC-1 cells. (50 µM: *** *p* = 0.0008; 100 µM: * *p* = 0.012 (adjusted P values, one-sample t-test) (**G**) Effect of pantoprazole on the different phases of the cell cycle in PANC-1 cells: G0/G1 (** *p* =0.0087; ** *p* =0.0013); S (NS); G2/M (* *p* =0.0113); paired t-test). Peak count analysis is shown in the right panel. Data are shown as means ± s.e.m. from the indicated number of independent experiments.

Next, we tested the effects of the proton pump inhibitors omeprazole and pantoprazole, and the potassium-competitive blocker SCH-28080, on cell proliferation on HPDE cells and the human PDAC cell lines BxPC-3, Capan-1, PANC-1 and MIA PaCa-2. Omeprazole and pantoprazole inhibit the gastric pump, whereas high concentrations of SCH-28080 can also act on the non-gastric H^+^, K^+^-ATPases [24]. Although both PPIs and P-CABs were developed to target the gastric H^+^, K^+^-ATPases, they also inhibit the non-gastric H^+^, K^+^-ATPases [17,25,26]. Short-term (24 h) incubation with the different PPIs applied individually or combined (omeprazole and SCH-28080) in normally buffered, not acidified culture media resulted in a dose-dependent decrease of BrdU incorporation in all cell lines processed (Figure 4A). PDAC cells were more responsive than HPDE cells, particularly to low concentrations of omeprazole and SCH-28080 (i.e., already at 1–10 µM). The combination of both drugs exerted a greater effect than either compound alone, decreasing the BrdU incorporation by approximately 70–80%. Since pantoprazole appears to raise intragastric pH and improves efficacy of chemotherapy in some solid tumors [21,27,28], we also investigated its effect on PANC-1 and MIA PaCa-2 proliferation. Treatment for 24 h with different concentrations of pantoprazole produced significant growth inhibition, especially in PANC-1 cells (Figure 4B), which was dose-dependent (Figure 4C). We further focused on PANC-1 cells as they have very high metabolism compared to other PDAC cells and one may expect highest H^+^ extrusion capacity [29]. Analysis of PANC-1 spheroids showed that the mean maximum cross-sectional area of the spheroids decreased by approximately 20% in pantoprazole-treated spheroids compared to controls (Figure 4D). Since it is possible that the antiproliferative effect of pantoprazole could be mediated by influencing other targets than the gastric H^+^,K^+^-ATPase, we also tested the effects of siRNAs against the *ATP4A* subunit on proliferation. Both siRNA-A and B reduced BrdU incorporation in PANC-1 cells by approximately 20% relative to the negative control (Figure 4E). However, the effect of siRNA-B did not remain statistically significant after correction for multiple comparisons (adjusted P = 0.0826). Since the siRNAs were designed to target only the HKα1 subunit, this may explain its lower efficiency on cell proliferation compared to the inhibitors that presumably affect both ATPases (see above). 

Since pancreatic ductal adenocarcinoma is characterized by a high metastatic potential, we also tested the impact of proton pump inhibitors on PDAC cell migration, which was significantly reduced by omeprazole, SCH-28080 and pantoprazole (Supplementary Figure S2). 

### 2.3. Effect of Pantoprazole on Cell Survival and Cell Cycle in PANC-1 Cells

To investigate whether the anti-proliferative effects observed with proton pump inhibitors were caused by cell death or cell cycle arrest, we assessed the potential cytotoxicity of pantoprazole by measuring lactate dehydrogenase (LDH) release in PANC-1 cells treated for 48 h with different concentrations of pantoprazole, using AT101 as a positive control (Figure 4F). Interestingly, less LDH was detected in the media of pantoprazole-treated cells compared to the control, indicating that the treatment did not diminish cell viability. In accordance, FACS analysis revealed that PPIs had very modest, if any, effects on cell death (Supplementary Figure S3). The lower LDH release with pantoprazole could possibly be due to the cytoprotective or growth inhibiting effect of pantoprazole on PANC-1 cells. Indeed, cell cycle analysis revealed that pantoprazole treatment increased the percentage of cells in G0/G1 phase and correspondingly decreased the G2/M phase, indicating significant cell cycle arrest (Figure 4G). 

### 2.4. Effect of Pantoprazole on H^+^ Transport, Membrane Potential and Cellular Signaling

**Figure 5:**
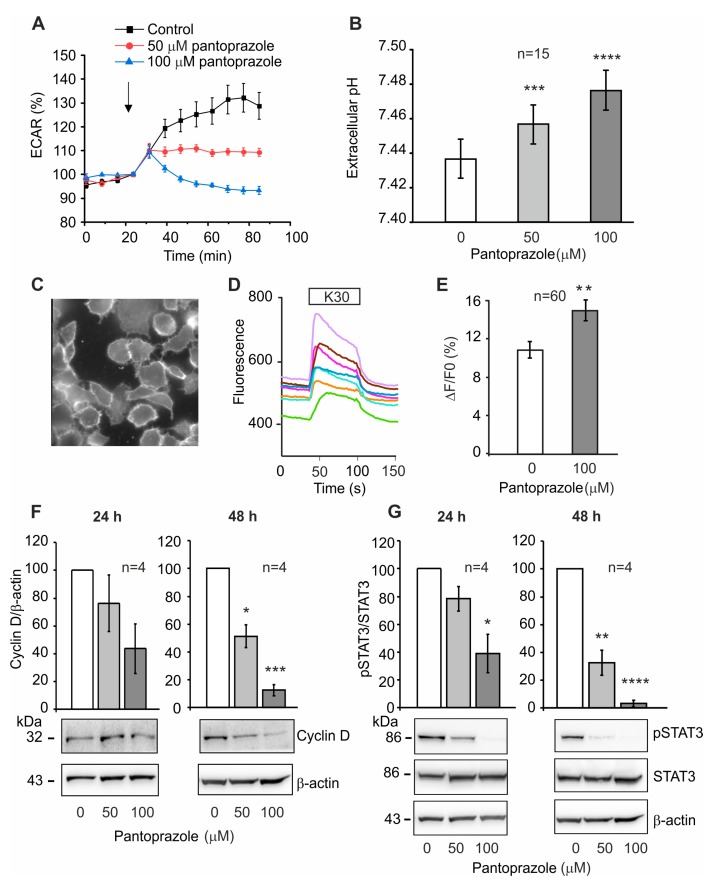
**Pantoprazole has fast effects on extracellular pH and membrane potentials in PANC-1 cells.** (**A**) Extracellular acidification rate monitored in Seahorse was normalized just prior to injection of control or pantoprazole solution. Extracellular acidification rate (ECAR) stabilizes within 15–20 min following “disturbance” due to injections. Representative experiment showing mean of 6 replicates ± s.e.m. (**B**) Readouts of extracellular pH measurements monitored by SNARF fluorescence (at 15 min) in control and pantoprazole treated cells. Pantoprazole increased extracellular pH in chronically and acutely treated cells (merged data for 24 h, 1 h and 15 min treatments). Data are shown as means ± s.e.m. from 15 experiments (50 µM: *** *p* = 0.0006, and 100 µM: **** *p* < 0.0001, paired t-tests). (**C**) Representative image of PANC-1 cells loaded with membrane voltage sensitive fluorophore VF2.1.Cl. (**D**) Typical response of several cells within one experiment in response to a 30 mM K^+^ step. (**E**) Depolarizing effect of K30 in control cells and those pretreated with pantoprazole for 15–20 min. Response in 60 cells in 10 independent experiments (** *p* = 0.0034 (unpaired t-test)). (**F**,**G**) Western blot quantification of cyclin D1 expression (normalized to β-Actin) and p-STAT3 (normalized to total STAT3 level) with representative blots. (**F**) Effect of pantoprazole on cyclin D1 expression after 24 and 48 h (48 h: 50 µM (*P = 0.0100) and 100 µM (*** *p* = 0.0002), one-sample t-test) and (**G**) STAT phosphorylation after 24 h (100 µM: * *p* = 0.0220) and 48 h treatment (50 µM: ** *p* = 0.0049 and 100 µM: **** *p* < 0.0001, one-sample t-test).

We hypothesized that part of the mechanisms that might induce cell cycle arrest could be related to PPIs effects on H^+^ transports and/or cell membrane potential. Figure 5A shows that pantoprazole (within 15 min) inhibits H^+^ extrusion of cancer cells to a low-buffered solution. In parallel experiments, pantoprazole increased extracellular pH (Figure 5B). Furthermore, using the voltage dye VF2.1.Cl (Figure 5C), we monitored PANC-1 cell membrane potential (V_m_) in response to a step in K^+^ from 4 to 30 mM in low-buffered solution. Normally, K^+^ step depolarizes pancreatic ducts by about 30 mV [30], which corresponds to about a 10% increase in VF2.1.Cl fluorescence (Figure 5D). Interestingly, pantoprazole increased the K^+^ step depolarizing response, indicating that the drug increased K^+^ conductance, G_K_, and thus hyperpolarized the V_m_ (Figure 5E).

Initial changes in pantoprazole-induced H^+^ and K^+^ transport (detected as pH and V_m_) could induce a longer lasting effect on cell signaling pathways, eventually leading to cell cycle arrest. Therefore, we evaluated the expression of cyclin D1 in PANC-1 cells treated with pantoprazole or control. Cyclin D1 was significantly decreased in a dose- and time-dependent manner following treatment with pantoprazole (Figure 5F). Moreover, pantoprazole had similar and parallel effects in down-regulating STAT3 activation (Figure 5G). 

### 2.5. Impact of Pantoprazole on Tumorigenesis in A Murine Orthotopic Xenograft Model

**Figure 6:**
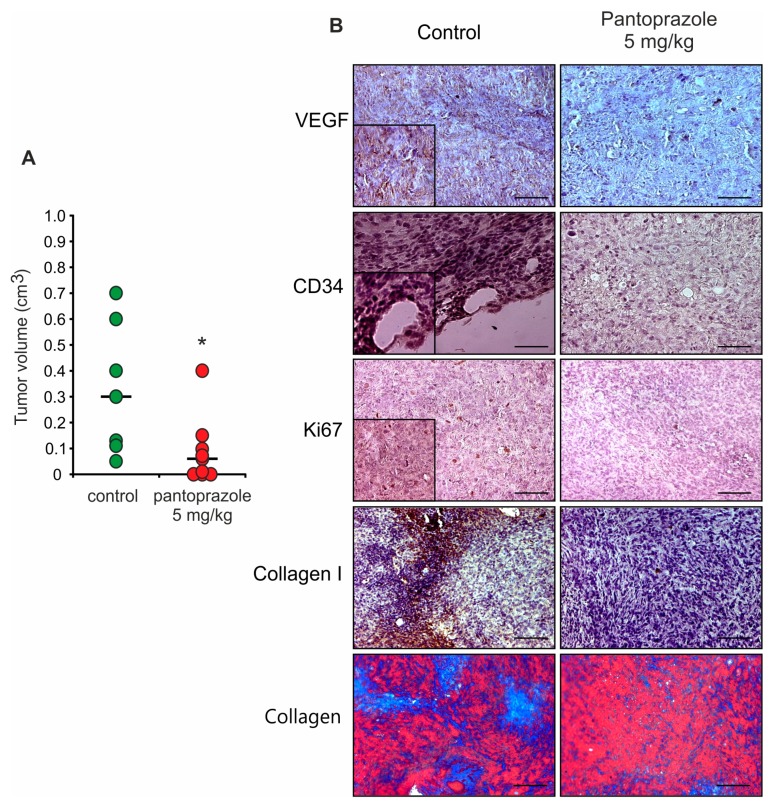
**PPI treatment reduces tumor size in the orthotopic mouse model of pancreatic cancer.** (**A**) Volume measurements of pancreatic tumor mass on mice transplanted with PANC-1 cells and treated with 5 mg/kg pantoprazole (red) or physiological saline solution (control, green). Horizontal bars show medians and circles are values for tumor size from individual mice. Asterisk indicates significant difference (* *p* = 0.0266; Mann Whitney test) between control and animals treated with pantoprazole. (**B**) Representative pictures of tumor paraffin sections from control and pantoprazole treated mice stained for vascular endothelial growth factor (VEGF) (Santa Cruz A-20, 1:100), CD34 (Cederlane, 1:100), Ki67 (Dako,1:50), collagen I (Abcam ab34710, 1:500) and collagen (Mallory’s Trichrome Connective Tissue Stain where collagen is stained with blue and nuclei with red). Inserts report a detail of the section, showing positivity for the VEGF, CD34, Ki67 proteins, acquired at higher magnification. Scale bars indicate 100 μm. For more thorough analysis of parameters measured and details about histology scores see Table 1.

Since our two- and three-dimensional in vitro cellular assays revealed that PPIs treatment affects PDAC cells, we tested whether this also applies to pancreatic tumor development in vivo in a murine orthotopic xenograft model. We used PANC-1 cells for the final xenograft model, as these cells could form three-dimensional (3D) spheroids and showed reliable responses to different concentrations of pantoprazole in the in vitro assays. For in vivo experiments, we treated the mice with 5 mg/kg pantoprazole, and Figure 6A shows that the tumor volume (cm^3^) was significantly reduced in the treatment group. Notably, all mice of the control group (7/7) developed measurable tumor sizes (tumor volume), while 70% of the animals in the pantoprazole treatment group (7/10) developed measurable sizes (Table 1). Regarding the tumor weight, only two animals in the treated group developed tumors with a weight that was detectable on the laboratory scale. For raw data see Table 1.

Slides obtained from a paraffin embedded specimen derived from three control mice and four mice treated with pantoprazole have been analyzed by IHC using antibodies against VEGF, CD34 and collagen I to identify the impact of the pantoprazole treatment on neoangiogenesis, and to evaluate tumor fibrosis (Figure 6B). Tissue sections from control mice were all positive for the VEGF, with strong positivity (score = 2–3, Table 1) for two of the three specimens, while mice treated with pantoprazole had tissues with a weak (score = 1) or negative (score = 0) immunostaining, as shown in the representative IHC fields (Figure 6B). Immunostaining for CD34 was moderate (score = 2) in all the control samples analyzed, whereas all specimen derived from mice treated with pantoprazole resulted in an absent or very poor signal (Figure 6B). Moreover, samples derived from pantoprazole-treated mice displayed weak or none Ki67 expression, a marker for proliferating cells, contrasting moderate staining in nuclei in tissue from control mice (Figure 6B). All control samples were positive for collagen I (score = 2), while a few or no positive cells have been detected in samples from pantoprazole treated mice (score = 0; 1). Additionally, Mallory staining showed decreased staining for collagen in specimens from treated animals (Figure 6B).

### 2.6. H^+^,K^+^-ATPase Expression and Function in Pancreatic Stellate Cells

**Figure 7:**
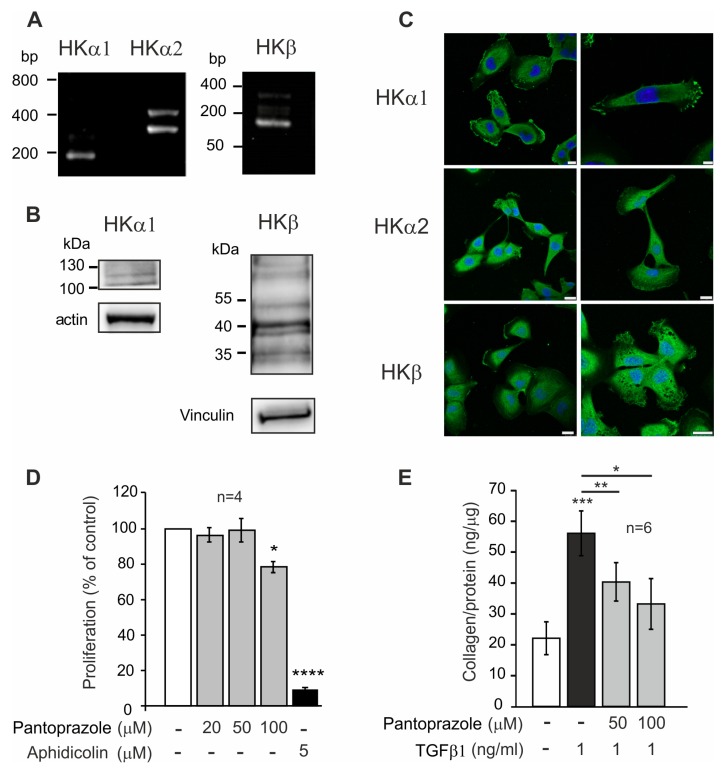
**Expression and function of H^+^, K^+^-ATPase in human pancreatic stellate cells PaSC.** (**A**) Representative gel of *ATP4A* (HKα1), *ATP12A* (HKα2) and *ATP4B* (HKβ) mRNA expression. (**B**) Representative western blot of HKα1 and HKβ protein expression. Loading controls were β-actin and vinculin. (**C**) Immunolocalization of HKα1 (Calbiochem 119101, 1:100), HKα2 (C384-M79, 1:300) and HKβ (Sigma A274, 1:100) is shown in green. DAPI (blue) was used to stain the nuclei. Scale bars indicate 25 µm. (**D)** Effect of pantoprazole on PaSC proliferation with 100 µM pantoprazole (* *p* = 0.0153) and Aphidicolin (5 µM) was used as a negative control (**** *p* < 0.0001), one sample t-test corrected for multiple comparison with Holm-Bonferroni method. (**E**) Effect of pantoprazole on collagen production induced by TGF-β1 (1 ng/ml) alone (*** *p* = 0.0002), or combined with pantoprazole 50 µM (** *p* = 0.0017) and 100 µM (* *p* = 0.0153), paired t-test. Data are shown as means ± s.e.m. from the indicated number of independent experiments.

Since our immunohistochemical analysis (Figure 1 and Figure 2) indicated that stromal cells in the tumor have reaction against H^+^,K^+^-ATPase antibodies, we investigated more closely whether pancreatic stellate cells (PaSC) also express these pumps. Here, we show for the first time that PaSC express transcripts and proteins for HKα1, HKα2 and HKβ (Figure 7A,B). The pumps are expressed on the plasma membrane, particularly at the ruffles/leading edges of single migrating cells (Figure 7C). Importantly, we show that pantoprazole is also functionally effective on PaSC, inhibiting their proliferation and collagen I secretion (Figure 7D,E).

## 3. Discussion

We have shown that neoplastic pancreatic tissue from mice and humans, as well as human pancreatic ductal adenocarcinoma (PDAC) cell lines and pancreatic stellate cells (PaSC), express H^+^,K^+^-ATPases. Treatment with proton pump inhibitors significantly inhibited PDAC cells and PaSC proliferation in vitro and tumor growth and fibrosis in the PDAC xenograft model in vivo. 

Pancreatic ducts are regarded as important players in PDAC development [31]. Normal human and rodent pancreatic ducts express H^+^,K^+^-ATPases, and these contribute to the physiological function, that is, pancreatic fluid secretion [14,15]. Pancreatic duct epithelium drives secretion by HCO_3_^−^ transport across the luminal membrane that is coordinated with H^+^ transport across the basolateral membrane. The H^+^ transport would give rise to periodic and local changes in pH that may have pathophysiological consequences [11,13]. Here, we show that pancreatic cancer cells also express gastric H^+^,K^+^-ATPase subunits HKα1 and HKβ and non-gastric HKα2 coded by *ATP4A*, *ATP4B* and *ATP12A.* First, this indicates their ductal phenotype, and second, it indicates that they have transporters necessary for H^+^ extrusion, enabling them to survive in the acidic tumor microenvironment and most likely contribute significantly to this acidosis. 

All H^+^,K^+^-ATPase subunits are expressed in the five human cancer cell lines that are commonly used as PDAC models (Figure 3). More importantly, we detected high expression of H^+^,K^+^-ATPase subunits in pancreatic samples obtained from murine pre-cancer and cancer models, as well as in human PDAC tissues (Figure 1 and Figure 2, Suppl. Figure S1). Notably, HK subunits staining, that was polarized to plasma membranes in normal pancreatic duct cells and agrees with previous studies [15], was now more intense and spread throughout PanINs, which comprise tall and disoriented cells in tortuous duct-like structures. In addition, pancreatic samples also disclosed a number of structures that represent acinar-to-ductal metaplasia (ADM) that also stain for HK subunits (Figure 1 and Figure 2). Intense staining was also detected in stromal fibrous cells, PaSC, which also express H^+^,K^+^-ATPases (Figure 7). This too may provide these cells with mechanisms to defend their intracellular pH in the acidic microenvironment and promote fibrosis, which is one of the hallmarks of pancreatic cancer. 

Using several proton pump inhibitors, as well as combination of these, we showed that the H^+^,K^+^-ATPases could be targeted pharmacologically, resulting in decreased PDAC proliferation capacity (Figure 4). This effect was not due to cytotoxicity of the drugs, but rather induction of the G0/G1 cell cycle arrest (Figure 4). A similar conclusion was reached in a recent study on Barrett´s esophagus cells, where omeprazole suppressed Hedgehog/Gli1 signaling and induced G0/G1 cell cycle arrest [32]. Other studies also showed that PPIs have anti-proliferative effects, inducing cell death often by apoptosis in different cancer cell types [18,20,21]. Most of these studies implemented high PPIs concentrations (e.g. >100 μM) and acidic or unbuffered media. It is widely believed that at high concentrations PPIs in fact inhibit the vacuolar V-ATPase, and older studies nicely show that these pumps do indeed require much higher concentrations of PPIs compared to H^+^,K^+^-ATPases [33,34]. V-ATPases are highly expressed in many cancers, including PDAC [35,36]. In mammalian cells, the most important cellular site for V-ATPase is in endocytotic and secretory granules. In certain specialized cells performing significant acid/base transport, e.g., renal intercalated cells, epididymal cells, osteoclasts and phagocytes, V-ATPase also localizes to the plasma membrane [37]. Additionally, one study shows that in MCF-7 cancer cells the pump re-localizes from lysosomes to the plasma membrane following lysosomal exocytosis [38], but this does not seem to be the case in PDAC [36]. 

Nevertheless, based on our study we propose that PPIs inhibited H^+^,K^+^-ATPases rather than V-ATPase for the following reasons. in vitro, in normal buffered solutions, PPIs were already effective at relatively low concentrations (1–50 μM), which are almost in the range of plasma concentrations (2–7 μM) of patients treated for peptic ulcers or gastroesophageal reflux disease with 20–40 mg/day of PPIs [27,39]. Taking into consideration dose conversion between mice and humans [40], also our in vivo administration of pantoprazole of 5 mg/kg would correspond to about 0.41 mg/kg human equivalent dose, giving around 25 mg/60 kg PPI per day used clinically. Further support is that the competitive potassium transport blocker, SCH-28080 and silencing of H^+^,K^+^-ATPases had similar effects as PPIs on in vitro PDAC cell functional assays (Figure 4). Most importantly, acute exposure to PPIs reduced H^+^ extrusion to extracellular space, increasing extracellular pH, and it increased K^+^ channel conductance, *G_K_* (Figure 5). These effects are in line with direct inhibition of cell membrane H^+^,K^+^-ATPases and resulting influence on pH-sensitive K^+^ channels. The latter candidates could be channels from the K2P family, such as alkaline activated TREK-1 channels that are expressed in PDAC cells [41]. 

Acute effects of pantoprazole at the plasma membrane (pH and/or *G_K_*) could be followed by cell cycle arrest. A similar effect of pantoprazole on cyclin D1 was reported for gastric adenocarcinoma [42]. The total amount of cyclin D1 is usually increased in cancer, and it allows the G1/S progression and therefore cell proliferation. As shown in Figure 4, pantoprazole causes cell cycle arrest in phase G0/G1, which is likely due to a reduction of cyclin D1 expression. Moreover, cyclin D1 is also a key factor for VEGF transcription, which is also lower in tumor masses from mice treated with pantoprazole (Figure 6). We also investigated the VEGF-dependent activation of the JAK/STAT (Janus kinase/signal transducer and activator of transcription) pathway, showing that STAT3 is correspondingly less activated with the drug treatment (Figure 5). This is in agreement with several recent studies on gastric cancer, which show that pantoprazole interfered with STAT3 signaling in different modes [43,44,45].

Two of the major reasons for poor prognosis of PDAC are early metastasis to other organs and resistance to drug delivery, due to fibrous and poorly perfused tumors. In our in vitro models we show that the major fibrosis-generating cells, PaSC, express the H^+^,K^+^-ATPases and that collagen secretion is sensitive to pantoprazole (Figure 7). 

Our findings from the in vitro models are unified in the murine in vivo pancreatic cancer model. The most important finding in our study is that PPIs administration (in clinically-relevant doses) to mice bearing pancreatic cancer model significantly decreased the tumor growth and fibrosis (Figure 6, Table 1). Furthermore, angiogenic/hypoxic and cell proliferative markers (VEGF, CD34 and Ki67) revealed negative or low expression of these in tissue obtained from pantoprazole-treated animals compared to the control animals (Figure 6). Moreover, collagen staining revealed less fibrotic tissue in treated animals, which is in line with our in vitro results showing that PaSC, expressing the H^+^,K^+^-ATPases, are sensitive to pantoprazole (Figure 7). 

Specific interference with tumor H^+^ dynamics (pH_i_ and pH_e_) and/or upregulated glycolytic metabolism may be an important step towards reversing the disrupted pH gradient and better treatment of solid tumors [9,46]. This approach has been made in a number of pre-clinical and also clinical studies by buffer/bicarbonate therapy [47,48], targeting pH regulating transporters and carbonic anhydrases [49] and pH-sensitive drug delivery systems [46]. Among these, PPIs are supposedly in the most advanced stages of clinical trials for prostate, colorectal and esophageal cancer [46,50], and it is presumed that they interact with cysteines of V-ATPases and other lysosomal and plasma membrane proteins. On the other hand, it should be noted that PPIs are widely used, and there is a valid concern about the harmful effect of long-term PPIs therapy [51], especially with respect to gastric and pancreatic carcinomas [52]. However, at least in gastric cancer, newer functional studies indicate that pantoprazole has anticancer effects via STAT3 regulated pathways (see above), opening a potential for safe anticancer drugs. Regarding the safety of long-term use of PPIs in relation to pancreatic cancer, epidemiological studies show different effects [53,54]. Even though the study on animal models indicates that PPIs can impair pancreatic exocrine secretion, there is a great reserve in pancreatic secretory capacity [14,15]. From the cancer perspective, the present study clearly shows that short-term administration of PPIs inhibits pancreatic H^+^, K^+^-ATPases and prevents tumor growth and fibrosis. Whether short-term systemic administration of PPIs to target pancreatic H^+^, K^+^-ATPases in pancreatic cancer cells and stellate cells could prove as a useful therapy will need to be assessed by careful controlled clinical studies. An alternative approach to target PPIs to pancreatic cancer microenvironment may prove a more appropriate strategy, given the apparent wide range of PPIs actions in many tissues.

## 4. Materials and Methods 

### 4.1. Chemicals

All standard chemicals were highest grades and purchased from Sigma-Aldrich unless otherwise stated. Omeprazole was dissolved in acidified ethanol (75% Ethanol and 1.5% 1M HCl), pantoprazole was dissolved in ddH_2_O and SCH-28080 in DMSO (Sigma D8418, ≥ 99.9%). 

### 4.2. Cell Culture

Human pancreatic ductal adenocarcinoma cells (from ATCC) and human pancreatic stellate cells (PaSC), RLT-PSC [55], were used in this study. AsPC-1 (RRID:CVCL_0152) and BxPC-3 (RRID:CVCL_0186) were grown in RPMI-1640 medium, Capan-1 (RRID:CVCL_0237) in Iscove’s Modified DMEM (IMDM), PANC-1 (RRID:CVCL_0480) and RLT-PSC in Dulbecco’s Modified Eagles Medium (DMEM) and MIA PaCa-2 (RRID:CVCL_0428) in DMEM/Ham’s F12, supplemented with 10% (or 20% for Capan-1) fetal bovine serum (FBS), 2.5% of horse serum for MIA PaCa-2 (Biochrom) and 1% penicillin/streptomycin. The human pancreatic duct epithelial cell line HPDE6-E6E7 (H6c7) (RRID:CVCL_0P38), which we refer to as HPDE, was obtained from Dr. Ming-Sound Tsao. HPDE cells were grown in KBM Basal Medium (Lonza, CC-3101). All cells were grown at 37 °C in a humidified atmosphere with 5% CO_2_. 

To grow spheroids, PANC-1 cells were seeded in ultra-low attachment round bottom 96-well plates (Corning), followed by centrifugation at 750 g for 15 min at 4 °C. Treatment with pantoprazole (50 μM) was initiated after 24 h and repeated after 4 days. After 7 days, bright field images of the spheroid’s largest cross-sectional area were taken in a Leica DMI6000B microscope. The areas of the maximum cross-sections were measured using Fiji software (http://fiji.sc/wiki/index.php/Fiji). 

### 4.3. RNA Extraction, RT-PCR and Real-time PCR

RT-PCR and real-time PCR were carried out as previously described [15]. Briefly, RNA from cultured cells was extracted using the RNeasy Mini Kit (Qiagen 74104) according to the manufacturer’s protocol. 1 μg RNA per reaction was used in OneStep RT-PCR Kit (Qiagen 210212). For real-time PCR, cDNA was synthesized using the RevertAid First Strand cDNA synthesis kit (Fermentas) and used as template for each PCR reaction. PCR reactions were run using Roche FastStart Universal SYBR Green Master with the following parameters: pre-incubation for 5 min at 95 °C, followed by 45 amplification cycles of 10 s at 95 °C, 1 min at 55 °C, and 30 s at 72 °C. The reactions were performed as triplicates, and β-actin, which has relatively stable expression in all cell lines tested, was used as a house-keeping gene. Table 2 shows primers used.

### 4.4. Western Blot

H^+^, K^+^-ATPase subunits detection was performed as described previously [15]. For signaling pathway analysis, PANC-1 cell lysate was boiled 3 min at 95 °C. Primary antibodies against gastric HKα1 (Abcam EPR12251, 1:1000), non-gastric HKα2 (C384-M79, 1:1000, kindly donated by J. J. H. H. M. De Pont and H. G. P. Swarts) [56], HKβ (Sigma A274, RRID:AB_258027, 1:1000), β-Actin (Santa Cruz Sc-47778, 1:1000, RRID: AB_626632), pSTAT3 (Cell Signaling #9145, 1:1000, RRID: AB_2491009), STAT3 (Cell Signaling #9139, 1:1000, RRID: AB_331757) and Cyclin D1 (Millipore #06-137, 1:500, RRID: AB_2070695) were used. 

### 4.5. Immunocytochemistry 

Cells were cultured on coverslips, fixed in 4% paraformaldehyde for 15 min at room temperature, then treated with 0.1 M TRIS-glycine (pH 7.4) for 15 min, permeabilized with 0.3% TritonX-100 for 15 min, blocked with 5–10% BSA for 30 min and incubated overnight at 4 °C with primary antibody against gastric HKα1 (Calbiochem 119101, RRID:AB_211408, 1:100), non-gastric HKα2 (1:300, C384-M79) and HKβ (1:100, A274, Sigma). Samples were incubated with appropriate secondary antibodies conjugated to Alexa 488 (Invitrogen, 1:400). DAPI (1:400, Molecular probes) was used as nuclear staining. Fluorescence was examined with 40× 1.3 NA or 63× 1.2 NA objectives in Leica TCS SP5 X confocal microscope (Leica Microsystems, Heidelberg). Images were analyzed in Leica software.

### 4.6. Immunohistochemistry 

Tissue sections from Pdx-1-Cre,LSL-KrasG12D/+ double mutant showing pre-cancerous pancreatic intraepithelial lesions (PanIN), and Pdx-1-Cre,LSL-KrasG12D/+, LSL-Trp53R172H/+ (KPC) triple mutant transgenic mice were deparaffinized and rehydrated according to standard procedures. Antigen retrieval was performed with 1x citrate buffer in 98 °C for 20 min. Tissue sections were treated with 3% H_2_O_2_ for 10 min and blocked with Blocking Buffer—Fish (BioFX, SurModics, Eden Prairie, MN, USA) for 20 min. Primary antibodies against gastric HKα1 (119101, Calbiochem), non-gastric HKα2 (C384-M79) and HKβ (A274, Sigma) were used at indicated concentrations. Slides were treated with N-Histofine Simple Stain Max PO anti-rabbit (Nichirei) for 1 h, stained with 3-Amino-9-ethylcarbazole (AEC) and counterstained with Mayer´s hematoxylin (Merck). Bright field pictures were taken with a Leica DMI6000B microscope.

Human pancreatic samples were from GeneTex (Irvine, CA, USA), or healthy parts of pancreas and PDAC resected from Whipple surgery. Samples were processed for immunostaining using similar protocols.

IHC on tumors obtained from nude mice bearing PANC-1-Luc was performed as previously reported [57]. Primary antibodies anti-VEGF-A (Santa Cruz, sc-152, 1:100, RRID: AB_2212984); anti-Ki 67 (Agilent (Dako), GA-626, 1:50, RRID: AB_2687921); anti-CD34 (Cedarlane, CL8927AP, 1:100, RRID: AB_10060158); anti-collagen I (Abcam, ab34710, 1:500, RRID: AB_731684) were used. To assess tumor fibrosis, samples were stained with Mallory´s trichrome connective tissue stain. Staining intensity is rated in a scale ranging from 0 to 3 (0 = negative, 1 = weak, 2 = moderate, 3 = strong). The scoring was estimated considering 0, no positive cells, 1 ranging between 1% and 30% of positive cells, 2 ranging between 31% and 60% of positive cells and 3 ranging between 61% and 100% positive cells. All the samples were evaluated using Leica DMR light microscope (Leica; Wetzlar, Germany).

### 4.7. Cell Proliferation 

Cells were plated on 96-well plates (COSTAR) and after 24 h the indicated concentrations of omeprazole, pantoprazole and SCH-28080 were added in culture media supplemented with 1% serum, followed by 24 h incubation, and assessment of the proliferation rate with Cell Proliferation ELISA, BrdU chemiluminescence kit (Roche) was made according to the manufacturer’s instructions. Luminescence was read in FLUOstar Optima (BMG, Labtech). 

### 4.8. siRNA Transfection

siRNAs targeting ATP4A (Silencer^®^ Select siRNAs, Ambion^®^: s1761; s1762, s1763, Invitrogen, Thermo Fisher Scientific) were used in a final concentration of 50 nM. The samples were analyzed relative to samples from cells treated with the non-targeting negative control No. 1 siRNA (Silencer® Select cat. number: 4390843, Invitrogen, Thermo Fisher Scientific). 

### 4.9. Cell Toxicity and Viability 

The In Vitro Toxicology Assay Kit, Lactate Dehydrogenase (LDH) (Sigma-Aldrich) was used to estimate the cytotoxicity of different concentrations of pantoprazole (20, 50 and 100 μM) and the apoptotic control AT-101 (10 μM) in PANC-1 cells, seeded in 96-well plates and treated for 48 h. Afterwards, cells were pelleted by centrifugation at 250 g for 4 minutes and the supernatant analyzed for LDH activity by absorbance measurement at 490/690 nm (FLUOstar Optima microplate reader (BMG, Labtech)).

Cell viability was estimated using flow cytometry and the Alexa Fluor^®^ 488 annexin V/Dead Cell Apoptosis Kit (Life Technologies). Cells were treated with the indicated concentrations of SCH-28080 and omeprazole for 24 h, harvested and stained with Annexin V and Propidium iodide according to the manufacturer’s protocol. A minimum of 20000 cells per sample were analyzed with FlowSight imaging flow cytometer (Merck-Millipore) and IDEAS software was used to calculate the percentage of live, apoptotic and necrotic populations. Electronic compensation was used to eliminate bleed through of fluorescence.

### 4.10. Cell Cycle Assays

PANC-1 cells were treated with indicated concentrations of pantoprazole for 24 h. Attached and floating cells were collected and fixed in ice cold ethanol for at least 2 h at 4 °C. Cells were stained for 20 min at 37 °C with 50 μg/ml Propidium iodide in presence of 50 μg/ml RNAse. A minimum of 50,000 cells per sample were analyzed by flow cytometry analysis of nuclear DNA content using a Calibur flow cytometer (BD biosciences, New Jersey, US) and CellQuest software (CellQuest Pro version 5.2.1 1994-2005, BD Biosciences, New Jersey, US). Gating was done using the software FlowJo^TM^ (v10.5.0, Becton, Dickinson and Company; USA).

### 4.11. Extracellular pH Determinations

PANC-1 cells in 96 well plates, pretreated with aphidicoline (5 μM), were incubated in low buffered Ringer solution (in mM: NaCl 143, KCl 4, CaCl_2_ 1.8, MgSO_4_ 0.8, NaH_2_PO_4_ 0.8, glucose 25, pH 7.4) and after 1 h equilibration, Dextran SNARF-1 (70,000 kDa, Molecular Probes D-3004, 50 μM) was added. Standard buffers of pH 6.0, 6.5, 7.0., 8.0 and 8.5 were treated similarly. Fluorescence at 590 nm and 520 nm was monitored after 490 nm excitation in FLUOstar Optima. PANC-1 cells were treated with pantoprazole 24 h or 1 h before experiment, and in one series pantoprazole was introduced together with Dextran SNARF-1. Fluorescence was read within 2 min for the next 120 min. In one series of experiments, extracellular acidification rate was monitored in Seahorse XF96 Analyzer (Agilent Technologies Denmark).

### 4.12. Membrane Voltage (V_m_) Measurements

Membrane voltage (*V_m_*) of PANC-1 cells was determined using voltage dye VF2.1.Cl, which was a kind gift from R. Tsien, using similar procedures as previously reported [41]. Briefly, cells were incubated with VF2.1.Cl (200 nM) for 20 to 40 min, washed and suspended in low buffered Ringer solution (see above) at 37 °C. Changes in *V_m_* were followed in Nikon Eclipse Ti microscope with a 40× NA1.4 objective. Fluorophore was illuminated 470 nm for 60 ms at 2 s intervals using a TILL Polychrome monochromator. Emission was collected at 500–570 nm by EMCCD camera (Andor X3 897) and digitized by FEI image processing system (Thermo Fischer Scientific). Changes in *V_m_* are presented as fluorescence (F), ΔF/F0 (%), where F0 represents the average value over the first minute and ΔF =F0 − F. Cells were not treated (controls) or pre-treated for 15–20 min with pantoprazole. We used a pulse of 30 mM KCl solution to evoke *V_m_* depolarization.

### 4.13. Collagen Release from Pancreatic Stellate Cells

PaSC cells were treated with 1 or 5 ng/ml of TGF-β1 (Sigma H8541) alone or in combination with indicated concentrations of pantoprazole, diluted in DMEM with no serum. After 24 h, the supernatant was collected and assayed for pro-collagen I α1/COLIA1 using the DuoSet ELISA kit for human Pro-Collagen I α1/COLIA1 (R&D Systems #DY6220).

### 4.14. Animal Experiments

Athymic nude mice (*Foxn1 nu*) supplied by Envigo RMS srl, San Pietro al Natisone (UD) were kept in controlled environmental conditions (22 °C, 55% humidity, 12 h light) and on a standard chow diet. Twenty female mice were injected orthotopically in the head of pancreas with 10^6^ PANC-1 cells tagged with luciferase (PANC-1-*luc*). Fifteen days after surgery, 5 mg/kg pantoprazole or saline (control) was administered I.P. every day for 21 days and bioluminescence was assessed. Two mice died during the experiment and one mouse after surgery. The remaining mice were sacrificed and tumors were weighted and size measured with calipers (4/3 π (a/2 × b/2 × c/2) if possible. Dissected tissues were processed for standard immunohistochemistry (see above). 

### 4.15. Ethics Approval and Consent to Participate

Samples of human pancreas from Whipple surgery were taken with the patient´s informed consent at Division of Oncological and Robotic General Surgery, Careggi University Hospital, Florence, Italy. All the procedures involving the animals were conducted according to the national and international laws on experimental animals (L.D. 26/2014; Directive 2010/63/EU) and to the approved experimental protocol procedure (Authorization N° 114/2016-PR).

### 4.16. Statistical Analyses

For in vitro experiments normalized data were analyzed with a one-sample t-tests, followed by correction for multiple comparisons with the Holm-Bonferroni method, when more than two different conditions were tested relative to the control. Non-normalized data were tested with a paired or an unpaired two-tailed Mann-Whitney test, t-test or a one-way ANOVA test with subsequent Bonferroni correction. in vivo experimental data were analyzed with a Mann-Whitney test. Software used for analyses, processing of data and preparation of graphs were: Excel; GraphPad Prism (version 7 and 8.4.0, GraphPad Software, La Jolla, CA, USA); OriginPro 9.1 (OriginLab Corporation, Northampton, MA, USA); and Corel Draw 2017.(version 19.1.0.419, Corel Corporation, Ottawa, Canada). *p* < 0.05 was accepted as significant. 

## 5. Conclusions 

In conclusion, our findings indicate that H^+^, K^+^-ATPases are promising targets in pancreatic ductal adenocarcinoma and that treatment with proton pump inhibitors or P-CABs could be a useful therapeutic strategy worthwhile of testing in a carefully controlled clinical settings. Furthermore, a better understanding of pH microenvironment in PDAC may provide additional targets and strategies to prevent tumor progression and metastases development.

## Figures and Tables

**Table 1:** Characteristics of pancreas tumor masses and histology.

Mouse n°	Group	Mass Weight (g)	Mass Volume (cm^3^)	Comments		Histology	
					VEGF	CD34	Ki67
1	Control	0.07	0.4	Enlarged spleen	-	-	-
6	Died before the end of the experiment	Died before the end of the experiment	Died before the end of the experiment	Died before the end of the experiment	Died before the end of the experiment	Died before the end of the experiment	Died before the end of the experiment
7	Control	Not detectable	0.05	-	3	2	2
8	Control	0.08	0.3	-	-	-	-
13	Died before the end of the experiment	Died before the end of the experiment	Died before the end of the experiment	Died before the end of the experiment	Died before the end of the experiment	Died before the end of the experiment	Died before the end of the experiment
14	Control	0.06	0.7	-	-	-	-
15	Control	0.07	0.6	-	-	-	-
17	Control	Not detectable	0.11	Small adhesion between pancreas and stomach	2	2	2
19	Control	Not detectable	0.133	Pancreas - stomach partially fused. 2 intestinal lesions	3	2	2
2	Pantoprazole	0.08	0.15	-	-	-	-
3	Pantoprazole	Not detectable	0.08	-	1	0	0
4	Pantoprazole	Not detectable	0.1	-	0	0	1
5	Pantoprazole	Not detectable	0.06	-	-	-	-
9	Pantoprazole	Not visible masses	Not visible masses	Two small lesions on the pancreas	-	-	-
10	Pantoprazole	Not detectable	0.1	-	1	0	0
11	Pantoprazole	No visible masses	No visible masses	Small lesion on the pancreas	-	-	-
12	Pantoprazole	0.09	0.4	Very small lesion of the stomach	-	-	-
16	Pantoprazole	No visible masses	No visible masses	-	-	-	-
18	Pantoprazole	Not detectable	0.07		1	0	1

The table summarizes the characteristics of pancreas tumor masses and histological results obtained from mice bearing PANC-1-Luc tumors that were not treated (control) or treated with pantoprazole (5 mg/kg). IHC experiments on tumor masses included three different markers (VEGF, CD34 and Ki67). Staining intensity is rated on a scale ranging from 0 to 3 (0 = negative, 1 = weak, 2 = moderate, 3 = strong). The scoring is estimated considering 0 no positive cells, 1 ranging between 1% and 30% of positive cells, 2 ranging between 31% and 60% of positive cells and 3 ranging between 61% and 100% positive cells.

**Table 2:** Primer sets used for RT-PCR and real-time PCR.

Genes	Primer Sequences
*ATP4A* (FW)	AAGATCTGCAGGACAGCTACGG
*ATP4A* (RW)	CTGGAACACGATGGCGATCA
*ATP12A* (FW)	CCCTGGGAGCTTTCCTTGTGTA
*ATP12A* (RW)	TTCCGAGGCCATAGGAGAGGAT
*ATP4B* (FW)	GGCCTTCTACGTGGTGATGAC
*ATP4B* (RW)	CCCGTAAACATCCGGCCTTA
*β-actin* (FW)	GTGACATTAAGGAGAAGCTGTGC
*β-actin* (RW)	CAATGCCAGGGTACATGGTG

## Supplementary Materials

The following are available online at https://www.mdpi.com/2072-6694/12/3/640/s1, Figure S1 Localization of H+/K+-ATPases subunits in human pancreas. (A) Images of human pancreas showing “normal” pancreatic ducts and (B) and PanINs (arrows). Samples were stained with HKα1 and HKα2 antibodies as in Fig. 2 (green) and nuclear stain (blue). Control images were taken in the absence of the primary or secondary antibodies. Scale bars indicate 50 μm. Representative images of pancreatic samples obtained from GenTex, Figure S2 PPIs decrease PDAC cells migration. Omeprazole (* *p* = 0.0134), SCH-28080 (* *p* = 0.0333) or combination of these (** *p* = 0.0052) were used to treat BxPC-3 (A) and Capan-1 (B) (Omeprazole: * *p* =0.0362, SCH-28080 * *p* = 0.0149, combination: ** *p* = 0.0017) cells and migration was determined in the Boyden chamber after 16-18 h. Cells were counted in at least seven fields per insert. Aphidicolin (5 μM) was added to stop cell proliferation. (C) Effect of Pantoprazole on migration of PANC-1 (50 μM: * *p* = 0.0277) and MIA PaCa-2 cells. Data shown above represent means ± s.e.m. obtained from a number of independent experiments indicated. Tested by one-sample t-test; *p* values were not corrected for multiple comparison as number of tests did not exceed two. For migration assays cells were seeded in the upper chamber of Boyden Chambers (transparent PET membrane, 8.0 μm pore size, Falcon) with aphidicolin (5 μM) and with/without indicated concentrations of omeprazole, SCH-28080 or pantoprazole in 1% serum media. The lower chamber contained 10 % serum media. After 16-18 h incubation cells were fixed in cold methanol and stained with Crystal Violet. Bright field images were taken with 10x objective in Leica DMI6000B microscope. Cells were counted by ImageJ, NIH, Figure S3 PPIs treatment has minimal effects on cell death in PDAC cells. (A) Representative dot plots of the three populations of BxPC-3 cells stained with Annexin V-(FITC)/propidium iodide (PI) and analyzed by flow cytometry following treatment with SCH-28080 alone and in combination with omeprazole. The green region contains the viable (Annexin V-/PI-) cell population, the purple region contains the apoptotic (Annexin V+/PI-) cells and the red region encloses the necrotic cells (Annexin V+/PI+). (B) Image gallery of representative cells for each population. (C) Quantification of live (green), early apoptotic (purple) and necrotic (red) populations expressed as percentage of cells. Data shown above represent means ± s.e.m. obtained from a number of independent experiments indicated. Asterisk indicates (* *p* = 0.0154, ANOVA test) and is referred to a statistical difference in the necrotic population compared to the control.
