# Supplementary material for: Proton Pump Inhibitors Reduce Pancreatic Adenocarcinoma Progression by Selectively Targeting H+, K+-ATPases in Pancreatic Cancer and Stellate Cells
Source: Cancers (Basel). 2020 Mar 10;12(3):640. doi: 10.3390/cancers12030640 (PMC7139746; doi:10.3390/cancers12030640)
Supplement: Supplementary file 1 [file cancers-12-00640-s001.pdf]

Supplementary Material

Proton pump inhibitors selectively target H^+,K^+-ATPases in pancreatic ductal adenocarcinoma

Marco Tozzi [1,#], Christiane E. Sørensen [1,2;#], Lara Magni [1], Nynne M. Christensen [1], Rayhana Bouazzi [1], Caroline M. Buch [1], Matteo Stefanini [3,4], Claudia Duranti [3], Annarosa Arcangeli [3] and Ivana Novak [1,*]

[1] Section for Cell Biology and Physiology, Department of Biology, University of Copenhagen, Copenhagen, Denmark
[2] Section for Clinical Oral Microbiology, Department of Odontology, Faculty of Health and Medical Sciences, University of Copenhagen, Denmark
[3] Department of Experimental and Clinical Medicine, Section of Internal Medicine, University of Florence, Florence, Italy
[4] DI.V.A.L. Toscana SRL, Florence, Italy
#shared authorship
* Correspondence: inovak@bio.ku.dk; Tel.: +45-3532-0275

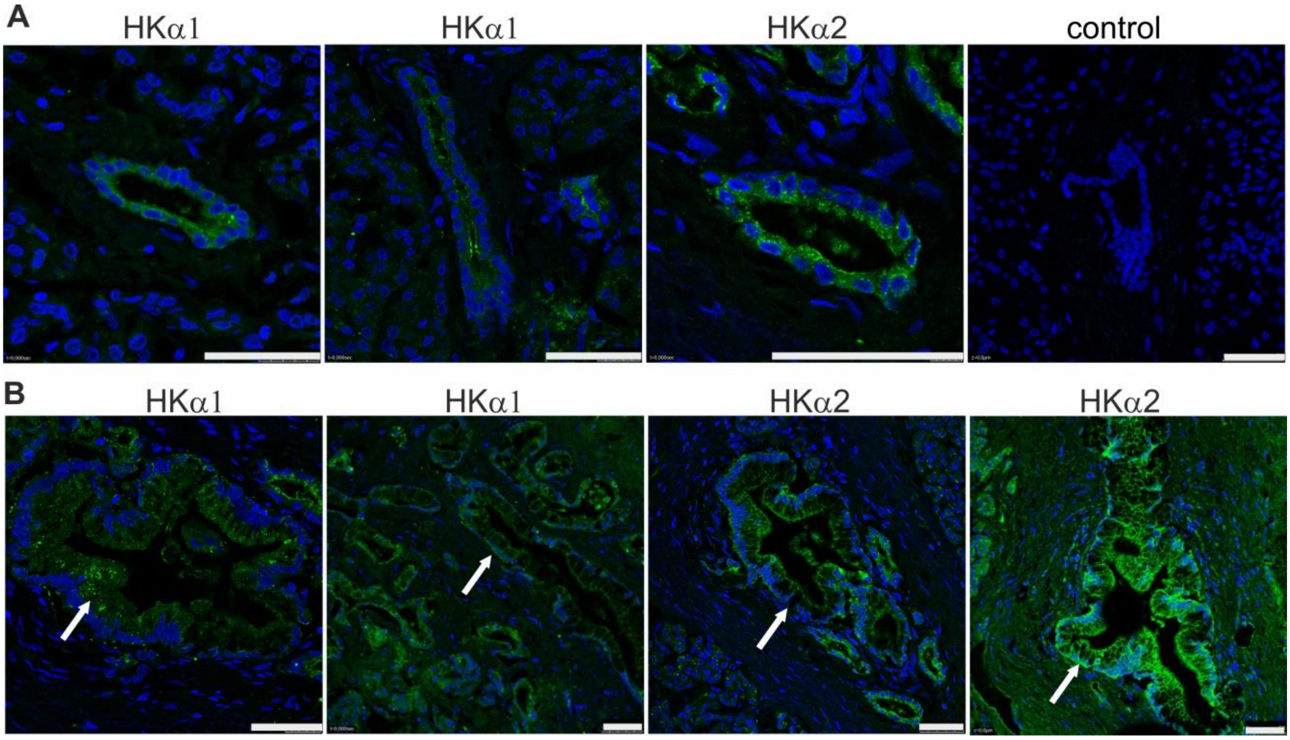

Supplementary Figure S1 Localization of H^+/K^+-ATPases subunits in human pancreas. (A) Images of human pancreas showing "normal" pancreatic ducts and (**B**) and PanINs (arrows). Samples were stained with HKα1 and HKα2 antibodies as in Fig. 2 (green) and nuclear stain (blue). Control images were taken in the absence of the primary or secondary antibodies. Scale bars indicate 50 µm. Representative images of pancreatic samples obtained from GenTex.

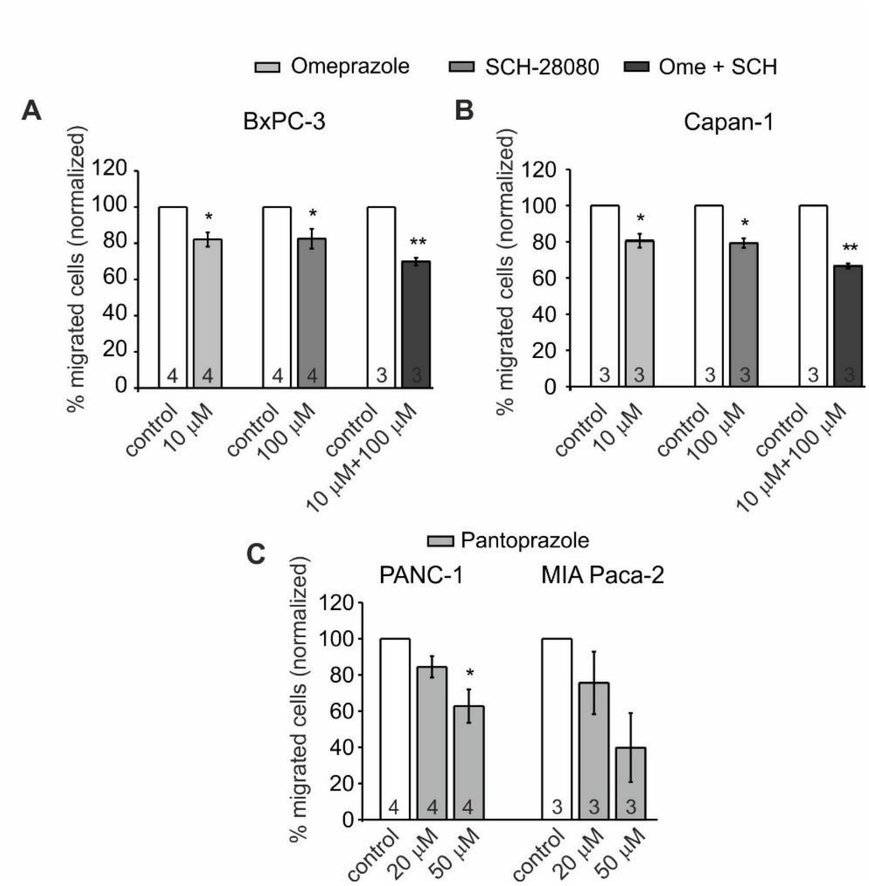

Supplementary Figure S2 PPIs decrease PDAC cells migration. Omeprazole (*P=0.0134), SCH-28080 (*P=0.0333) or combination of these (**P=0.0052) were used to treat BxPC-3 (**A**) and Capan-1 (**B**) (Omeprazole: *P=0.0362, SCH-28080 *P=0.0149, combination: **P=0.0017) cells and migration was determined in the Boyden chamber after 16-18 h. Cells were counted in at least seven fields per insert. Aphidicolin (5 µM) was added to stop cell proliferation. (**C**) Effect of Pantoprazole on migration of PANC-1 (50 µM: *P=0.0277) and MIA PaCa-2 cells. Data shown above represent means ± s.e.m. obtained from a number of independent experiments indicated. Tested by one-sample t-test; P values were not corrected for multiple comparison as number of tests did not exceed two. For migration assays cells were seeded in the upper chamber of Boyden Chambers (transparent PET membrane, 8.0 µm pore size, Falcon) with aphidicolin (5 µM) and with/without indicated concentrations of omeprazole, SCH-28080 or pantoprazole in 1% serum media. The lower chamber contained 10 % serum media. After 16-18 h incubation cells were fixed in cold methanol and stained with Crystal Violet. Bright field images were taken with 10x objective in Leica DMI6000B microscope. Cells were counted by ImageJ, NIH.

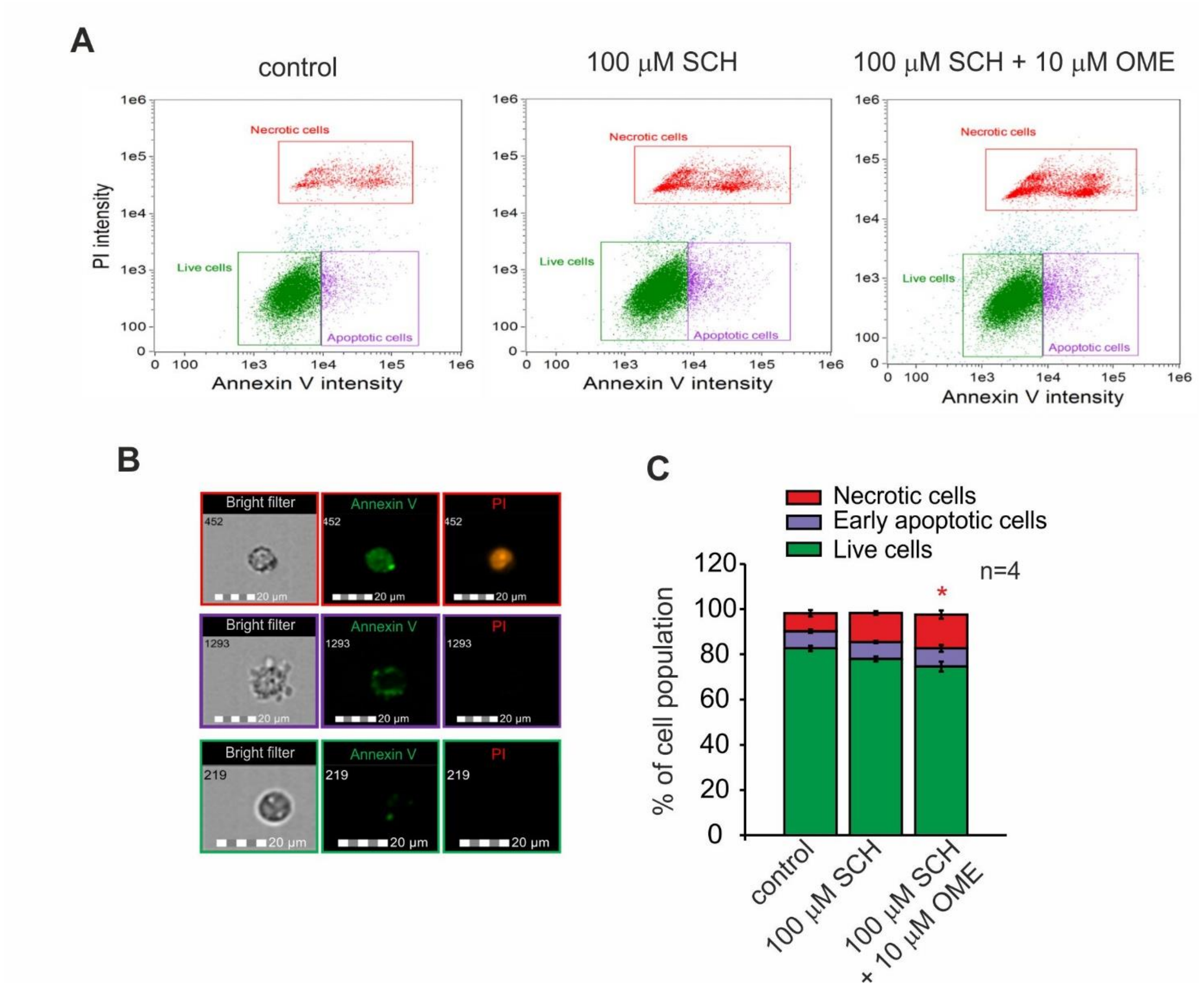

Supplementary Figure S3 PPIs treatment has minimal effects on cell death in PDAC cells. (**A**) Representative dot plots of the three populations of BxPC-3 cells stained with Annexin V-(FITC)/propidium iodide (PI) and analyzed by flow cytometry following treatment with SCH-28080 alone and in combination with omeprazole. The green region contains the viable (Annexin V^- /PI^-) cell population, the purple region contains the apoptotic (Annexin V^+/PI^-) cells and the red region encloses the necrotic cells (Annexin V^+/PI^+). (**B**) Image gallery of representative cells for each population. (**C**) Quantification of live (green), early apoptotic (purple) and necrotic (red) populations expressed as percentage of cells. Data shown above represent means ± s.e.m. obtained from a number of independent experiments indicated. Asterisk indicates (*P=0.0154, ANOVA test) and is referred to a statistical difference in the necrotic population compared to the control.
